# Don’t forget the trunk in Duchenne muscular dystrophy patients: more muscle weakness and compensation than expected

**DOI:** 10.1186/s12984-019-0515-y

**Published:** 2019-03-27

**Authors:** L. H. C. Peeters, I. Kingma, J. H. van Dieën, I. J. M. de Groot

**Affiliations:** 1 Department of Rehabilitation, Radboud University Medical Center, Donders Centre for Neuroscience, P.O. Box 9101, Nijmegen, HB 6500 The Netherlands; 2 Department of Human Movement Sciences, Faculty of Behavioral and Movement Sciences, Vrije Universiteit Amsterdam, Amsterdam Movement Sciences, Amsterdam, The Netherlands

**Keywords:** Muscular dystrophy, Kinematics, Electromyography, Trunk, Activities of daily living

## Abstract

**Background:**

Performing daily activities independently becomes more difficult in time for patients with Duchenne muscular dystrophy (DMD) due to muscle weakness. When performing seated daily activities, the trunk plays an indispensable role besides the upper extremities. However, knowledge is lacking on the interaction between trunk and upper extremities. Therefore the aim was to investigate whether patients with DMD use trunk movement to compensate for reduced arm function when performing seated tasks, and whether this is related to increased muscle activity.

**Methods:**

Eighteen boys with DMD and twenty-five healthy controls (HC) performed several tasks when sitting unsupported, like reaching (and placing) forward and sideward, drinking and displacing a dinner plate. Maximum joint torque and maximum surface electromyography (sEMG) were measured during maximum voluntary isometric contractions. Three-dimensional movements and normalized sEMG when performing tasks were analyzed.

**Results:**

Significantly decreased maximum joint torque was found in DMD patients compared to HC. Trunk and shoulder torques were already decreased in early disease stages. However, only maximum trunk rotation and shoulder abduction torque showed a significant association with Brooke scale. In all reaching and daily tasks, the range of motion in lateral bending and/or flexion-extension was significantly larger in DMD patients compared to HC. The trunk movements did not significantly increase with task difficulty (e.g. increasing object weight) or Brooke scale. Normalized muscle activity was significantly higher in DMD patients for all tasks and muscles.

**Conclusions:**

Boys with DMD use increased trunk movements to compensate for reduced arm function, even when performing relatively simple tasks. This was combined with significantly increased normalized muscle activity. Clinicians should take the trunk into account when assessing function and for intervention development, because DMD patients may appear to have a good trunk function, but percentage of muscle capacity used to perform tasks is increased.

**Electronic supplementary material:**

The online version of this article (10.1186/s12984-019-0515-y) contains supplementary material, which is available to authorized users.

## Background

For patients with Duchenne muscular dystrophy (DMD), performance of daily activities becomes more difficult over time due to progressive muscle weakness. DMD is an x-linked neuromuscular disorder with an incidence of approximately 1 in 6000 live male births [1]. Mean loss of ambulation is around 11 years with use of corticosteroids in the Netherlands [2], but patients report difficulties in performing daily activities involving arm movements already earlier [3].

Decreased upper extremity function is already visible in early stages of DMD and precedes the decline in activity performance [4]. Trunk weakness seems to occur in later disease stages. Trunk function seems relatively good and stable in the ambulatory phase, but starts to decrease when boys become non-ambulant [5, 6]. However, both measures used (Segmental Assessment of Trunk Control [5] and Motor Function Measure [6]) are influenced by upper extremity function too and therefore might not completely represent trunk function alone.

Knowledge concerning the relation between upper extremity movement and trunk movement in patients with DMD is completely lacking at present [7]. In healthy adults and children, coordination of upper extremity and trunk motion is essential for accomplishing daily tasks [8, 9]. For DMD patients this may be even more, because clinically they show increased trunk movement to compensate for reduced arm function. Understanding the use of compensatory trunk movements could be beneficial for the development of interventions, such as physical exercise training, seating adjustments and assistive technology.

Therefore, the aim of this study was to investigate how DMD patients use trunk movement to compensate for reduced arm function. We hypothesise that compensatory trunk movement is dependent on task difficulty, disease progression and related to increased trunk muscle activity.

## Methods

### Participants

Eighteen male DMD patients and twenty-five healthy controls (HC) (13 males) participated in this study. Participants were included if they were between 6 and 21 years of age, able to show arm motor skills at request and could sit independently (without back or arm rests) for at least 10 min. DMD patients needed to have a genetically confirmed diagnosis of DMD. Participants were excluded if they had (other) diseases affecting the arm, trunk or head movements, and if they had received spinal fusion surgery.

DMD participants were recruited through advertisements by two patient organizations (Duchenne Parent Project and Spierziekten Nederland) and through the outpatient clinic of the Radboudumc in Nijmegen. HC were recruited from local primary schools, high schools and university. Prior to participation, written informed consent was given by participants when over 12 years old, and by the children’s parents or guardians for all participants younger than 18 years old. The study was approved by the medical ethics committee Arnhem-Nijmegen (NL53143.091.15) and all data were handled according to the guidelines of good clinical practice.

### Procedures

We used the same procedure as the one employed in a previous study with healthy children [10]. All participants were seated on a height adjustable chair with a multi-celled air cushion (Starlock, Star Cushion Products, Freeburg, IL), without back- or armrests. The sitting height was adjusted so that the knees were flexed 90° and both feet were flat on the ground.

First, to determine maximum trunk range of motion, participants were asked to perform a maximum active flexion movement of their trunk from a seated position, immediately followed by a maximum active extension movement of their trunk (keeping both feet on the ground). The same was done for maximum axial rotation and lateral bending. Thereafter, a series of tasks were performed with the dominant hand at a self-selected speed. Several reaching (and placing) tasks were performed at shoulder height: reaching forward, sideways and contra-laterally at a 45 degrees angle in the transverse plane. Participants had to touch a reference frame positioned at the desired position, or to place an object on the reference frame. Reaching distance and object weight were varied, resulting in the following combinations for forward, lateral and contra-lateral reaching: nearby-0 g (“N-0”), nearby-500 g (“N-500”), far-0 g (“F-0”). Contra-lateral reaching was not performed at a far distance. Nearby was defined as the distance that could be reached by stretching the arm (i.e. 100% arm length for HC, but could be closer for DMD) and far as 133% of arm length when possible, otherwise as maximum reaching distance. Arm length was defined as the distance from mid-acromion to the centre of the hand. Furthermore, subjects were asked to perform two daily tasks: displace a porcelain plate (circa 600 g) from left to right on a table with both hands (“Plate”) and bring a cup of 200 g to the mouth (“Drink”). The drink task was based on the instructions of the Performance of the Upper Limb [11]. No instructions were given on how to perform the tasks.

### Outcome measures

#### Participant characteristics

The following participant characteristics were noted based on self-reports: age, weight, height, arm preference, age of diagnosis (if applicable), use of corticosteroids, wheelchair confinement, pain in upper body at time of participation and occurrence of scoliosis. Sitting height was measured and, for DMD patients, the Vignos lower extremity scale [12] and Brooke upper extremity scale [13] were used for clinical assessment of leg and arm function, respectively.

#### Three dimensional motion analysis

We used the same data acquisition and analysis as employed in a previous study with healthy children [10]. An optical motion capture system (Vicon, Oxford, UK) was used to record 25 single reflective markers, which were placed on the skin to define positions and orientations of the head, trunk, pelvis and both arms during task performance. The markers divided the trunk initially into four segments (upper thoracic, lower thoracic, upper lumbar and lower lumbar), because the trunk cannot be seen as rigid segment [10]. However to make the data more concise, we decided to report here the trunk movement as one segment (i.e. summation of the trunk and pelvis segment angles). Distribution of movement patterns over the individual trunk segments was essentially the same among HC and DMD.

All kinematics data were filtered with a bi-directional 4th order Butterworth low-pass filter (cutoff frequency of 6 Hz). Trunk joint angles are expressed relative to the global coordinate system, and are described in all three movement directions: flexion-extension (i.e. sagittal plane), lateral bending (i.e. frontal plane) and axial rotation (i.e. transversal plane).

Maximum trunk joint angles in all three movement directions were determined when performing the active range of motion (ROM) tasks for trunk. For the reaching tasks and daily tasks, the trunk ROM between the start and end of the task was determined. Start and end of a task were defined as the time where the velocity of the wrist exceeded/got below 5% of its peak velocity. Direction of the movement was defined for all reaching tasks by subtracting the trunk joint angle at the time of touching the reference frame, from the joint angle at the start. This defined whether the movement was in a positive or negative direction (i.e. flexion or extension, or towards dominant or non-dominant side). Towards dominant side reflects the side of the hand used to perform the tasks.

#### Joint torque and surface electromyography

Muscle activity was measured with the use of surface electromyography (sEMG) (Zerowire EMG, Aurion, Italy) and was recorded with a sample frequency of 1000 Hz. Electrodes were placed on the following muscles on both sides of the body: iliocostalis (6 cm from spinous processes of the 1st lumbar vertebrae), longisimus (3 cm from spinous processes of the 3rd lumbar vertebrae), external oblique (3 cm from axillae midline at height of umbilicus), trapezius descendens (1/2 on the line from the acromion to the spinous processus of the 7th cervical vertebrae) and medial deltoid (1/3 on the line from acromion to lateral epicondyle of the elbow) [14]. The trapezius and deltoid muscles were included to get an estimate of shoulder muscle effort when performing tasks. Electrodes on the iliocostalis muscle were not placed on the smaller participants (*n* = 9), due to space limitations on the back.

Maximum force was measured using a static frame myometer. The frame consisted of a KAP-E Force Transducer (range 0.2–2000 N) (Angewandte System Technik, Dresden, Germany) and a height and position adjustable frame (custom made at the VU medical centre, Amsterdam, the Netherlands). The force signal was filtered with a bi-directional 4th order low-pass filter of 30 Hz. Afterwards the measured maximum force signal was converted to joint torque by multiplying the force with the segment length (i.e. moment arm) and additionally resulting torques were also corrected for body weight.

Maximal voluntary isometric contractions (MVIC) were performed to determine maximal joint torques and corresponding sEMG amplitudes. Participant’s positions for MVIC measurements were adapted to seated positions so all participants with DMD could perform the measurements. Participants performed two MVIC tasks for each of the following movements: trunk flexion, trunk extension, lateral bending trunk (left and right), shoulder elevation (left and right) and shoulder abduction (left and right). Participants were encouraged to push as hard as they could for 3 s. When the maximum force of the MVIC tasks varied more than 10% between the two trials, an additional trial was recorded. A 4th order Butterworth filter (20–450 Hz) was used to filter the sEMG signals, followed by rectification and low-pass filtering (3 Hz) of the signals to obtain the linear envelopes.

The maximum sEMG amplitude for each trunk muscle was taken as the highest amplitude from the four MVIC tasks of the trunk, for the trapezius as the highest amplitude from the shoulder elevation task and deltoid as the highest amplitude from the shoulder abduction task.

Normalized sEMG amplitudes were used to describe the percentage of muscle capacity used during task performance. These were calculated by dividing the sEMG amplitudes during task performance, by the corresponding maximum sEMG amplitudes. Subsequently, average muscle activity of the back muscles (i.e. longissimus and iliocostalis both sides) and average activity of the abdominal muscles (i.e. external oblique both sides) were calculated. If there were more than two missing values, i.e. trials that failed due to inability of the participant to perform the task, or technical errors such as missing signals due to loose electrodes, the average muscle activity was defined as missing value.

All analyses were performed using Matlab R2014b (Math Works, USA) software.

### Statistics

Non-parametric tests were used since most of the data were not normally distributed. Median values and interquartile ranges were used to describe the participant characteristics. Wilcoxon rank sum test was used to assess differences between DMD patients and HC and the Kruskal-Wallis test to assess differences between DMD patients with different scores on the Brooke scale.

To test the hypotheses that compensatory trunk ROM would increase with task difficulty, trunk ROM when reaching without weight was subtracted from trunk ROM when reaching with 500 g object for each individual. Afterwards the change in trunk ROM between DMD patients and HC was assessed with the Wilcoxon rank sum test.

The range of motion is depicted in graphs, where the boxes represent 25th, 50th and 75th percentile, whiskers minimum and maximum non-outlier values and dots indicate outliers (greater than 1.5 times the interquartile range). All statistical analyses were performed using Matlab R2014b (Math Works, USA) and the statistical significance level was set at *α* = 0.05.

## Results

### Subject demographics

Participant characteristics are described in Table 1. Of the corticosteroid users, three patients used Deflazacort and the others used Prednisolone. The Vignos classifications of the DMD participants were: 1 (*n* = 1), 2 (n = 1), 3 (n = 1), 4 (n = 1), 5 (*n* = 2), 7 (n = 2) and 9 (*n* = 9); and the Brooke classifications: 1 (*n* = 6), 2 (n = 6), 3 (*n* = 5). One DMD participant left the assessment before the protocol was finished and was therefore excluded from the analysis. Termination of the measurement was unrelated to the protocol or measurement itself.

**Table 1:** Participant characteristics

	Healthy			DMD		
	n	median	IQR	n	median	IQR
Age [years]	25	13.2	[9.4–18.0]	17	13.1	[11.7–15.8]
Gender [male/female]	13/12			17/0		
Weight [kg]	25	48.6	[30.5–63.5]	15	48.0	[40.0–51.5]
Height [cm]	25	160.0	[136.5–171.0]	15	150.0	[145.5–157.0]
Sitting height [cm]	25	62	[50.5–65.6]	12	50.0	[47.8–56.3]
Pain at time of participation [n]	0			0		
Age of diagnosis [years]				16	4	[3–5]
Corticosteroid use [n]	0			15		
Wheelchair confinement indoors [n]	0			10		
Wheelchair confinement outdoors [n]	0			14		
Scoliosis [n]	0			2		

### Active range of motion and joint torque

**Fig. 1:**
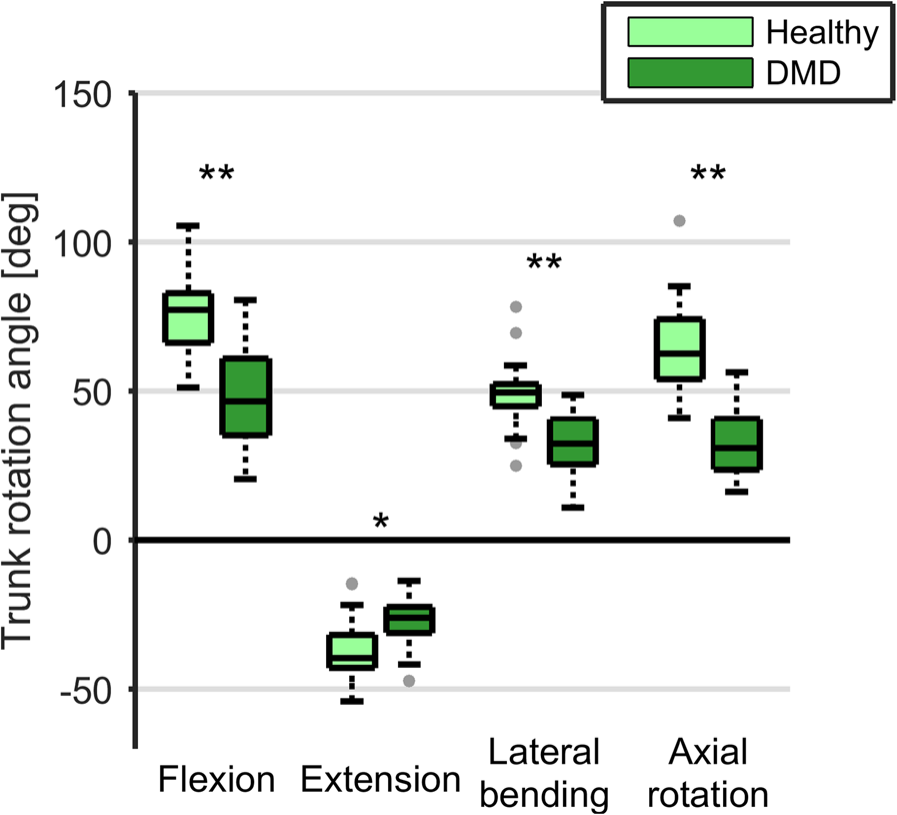
Maximum trunk angle during active trunk movements. *p < 0.05, **p < 0.01

The maximum trunk angles were significantly lower (*p* < 0.05) in all movement directions for DMD patients compared to HC (Fig. 1). Only trunk axial rotation showed a significant relation (*p* = 0.014) with Brooke scale, where smaller angles were seen with a higher Brooke scale (Additional file 1).

DMD patients had significantly (*p* < 0.01) lower joint torques compared to HC in all muscle groups and tasks both with and without normalization to body weight (Table 2). However, a significant (*p* < 0.05) effect of Brooke scale was only found when correcting the joint torques for body weight, except for the trunk extension torque (Table 2, Additional file 2). Both with and without correction for body weight, trunk torque was already approximately two times smaller in DMD patients with Brooke scale 1 compared to HC.

**Table 2:** Maximum joint torques (in Nm and Nm/kg) of trunk and shoulder

	Healthy			DMD			*p*-value HC vs DMD	*p*-value Brooke scale
	n	median	IQR	n	median	IQR		
Joint torque [Nm]								
Trunk (flexion)	25	47.5	[24.5–58.2]	17	20.3	[14.8–25.0]	**0.001**	0.120
Trunk (extension)	25	43.9	[19.8–78.6]	16	21.4	[14.3–30.8]	**0.003**	0.673
Trunk (lateral bending)	25	44.4	[27.5–65.2]	17	23.6	[17.0–33.1]	**0.001**	0.355
Shoulder elevation	25	50.0	[30.1–95.8]	17	18.2	[12.6–24.4]	**< 0.001**	0.244
Shoulder abduction	25	30.6	[20.0–46.9]	17	11.9	[4.4–14.8]	**< 0.001**	**0.019**
Joint torque [Nm/kg]								
Trunk (flexion)	25	0.94	[0.73–1.09]	15	0.43	[0.34–0.52]	**< 0.001**	**0.009**
Trunk (extension)	25	0.81	[0.72–1.25]	14	0.41	[0.31–0.52]	**< 0.001**	0.585
Trunk (lateral bending)	25	0.99	[0.88–1.14]	15	0.45	[0.39–0.64]	**< 0.001**	**0.014**
Shoulder elevation	25	1.10	[0.9–1.76]	15	0.34	[0.26–0.56]	**< 0.001**	**0.027**
Shoulder abduction	25	0.69	[0.61–0.8]	15	0.2	[0.08–0.28]	**< 0.001**	**0.012**

### Performing daily activities

**Fig. 2:**
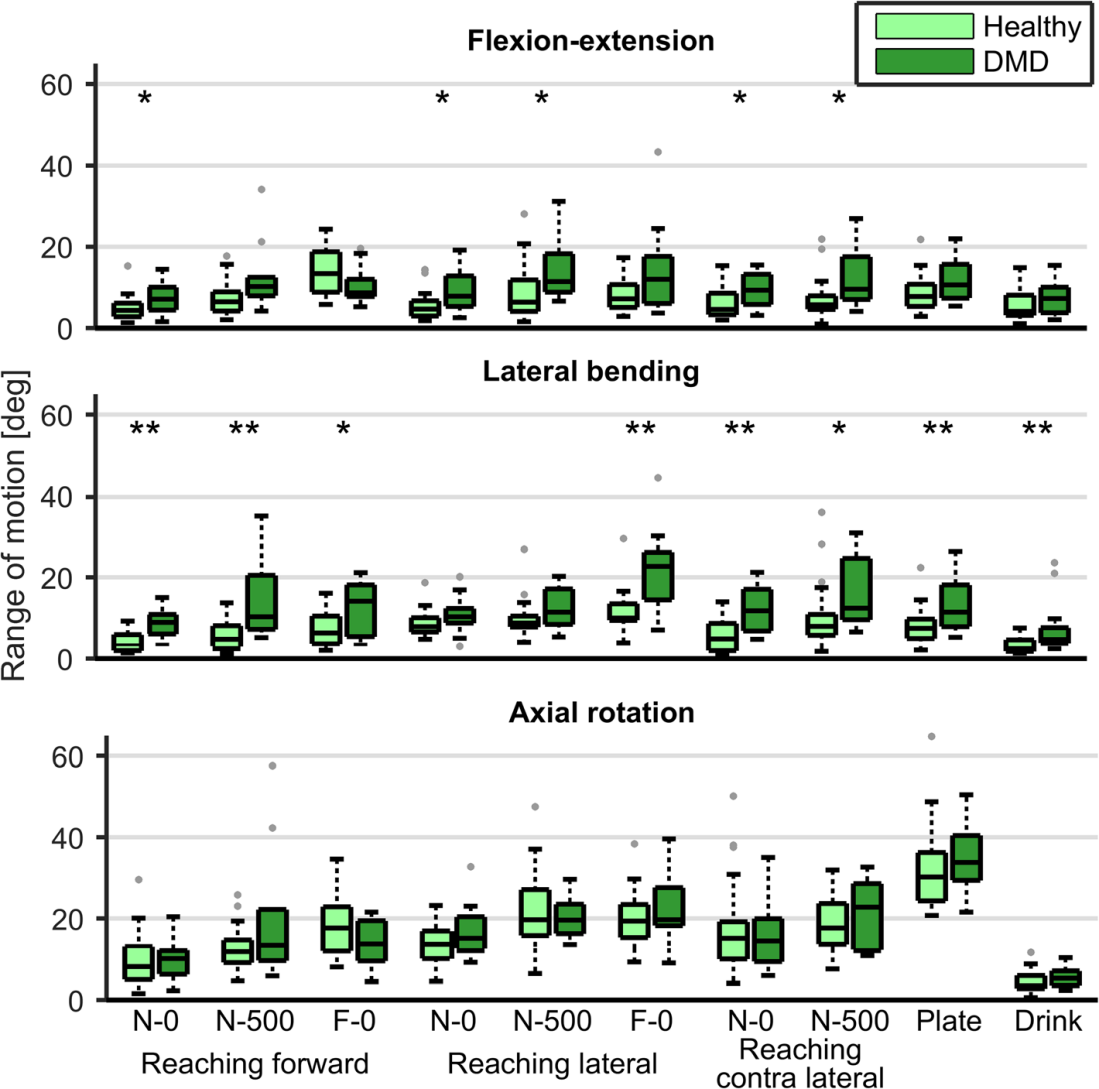
Trunk ROM in DMD patients and healthy controls when performing daily activities. Abbreviations: N = near, F = far, 0 = without weight, 500 = 500 g object, * *p* < 0.05, ** *p* < 0.01

Trunk ROM in one or more movement directions was significantly higher in DMD patients compared to HC in all of the reaching and daily tasks (Fig. 2, Additional file 3). Increased lateral bending was seen for all tasks, except for reaching laterally at nearby distance, and increased trunk flexion-extension was seen for most of the nearby reaching tasks, i.e. reaching at arm length distance. However, the change in trunk ROM with task difficulty (e.g. object weight), was not significantly different between DMD patients and HC (Table 3), except when reaching forward were DMD patients used significantly more trunk flexion-extension movement compared to HC. The change in trunk axial rotation tended to be higher in DMD patients when reaching forward (*p* = 0.061) and contra-laterally (*p* = 0.062).

**Table 3:** Change in trunk ROM (in degrees) between reaching with 500 g and without weight

Trunk ROM direction	Reaching direction	Healthy			DMD			*p*-value HC vs DMD
		n	median	IQR	n	median	IQR	
Flexion-extension	Forward	25	8.5	[3.2–21.1]	9	26.6	[13.4–31.1]	**0.017**
	Sideward	25	5.8	[2.5–9.8]	8	11.6	[5.1–21.8]	0.303
	Contra-lateral	25	5.6	[2.5–10.8]	8	11.7	[4.1–18.0]	0.231
Lateral bending	Forward	25	4.1	[1.2–8.3]	9	7.3	[2.1–16.8]	0.458
	Sideward	25	7.5	[5.3–11.4]	8	15.6	[4.7–21.8]	0.284
	Contra-lateral	25	3.0	[−2.0–5.0]	8	8.4	[0.4–16.2]	0.125
Axial rotation	Forward	25	4.7	[1.6–6.6]	9	11.0	[3.2–16.5]	0.061
	Sideward	25	8.6	[3.6–15.3]	8	8.4	[3.7–10.7]	0.850
	Contra-lateral	25	5.0	[−2.9–9.0]	8	11.4	[5.8–14.5]	0.062

A significant increase in trunk ROM with Brooke scale was only found for the drinking task (in the frontal plane) (*p* = 0.007) and when reaching contra-lateral with 500 g object (in the frontal plane) (*p* = 0.025) (Additional file 3).

**Fig. 3:**
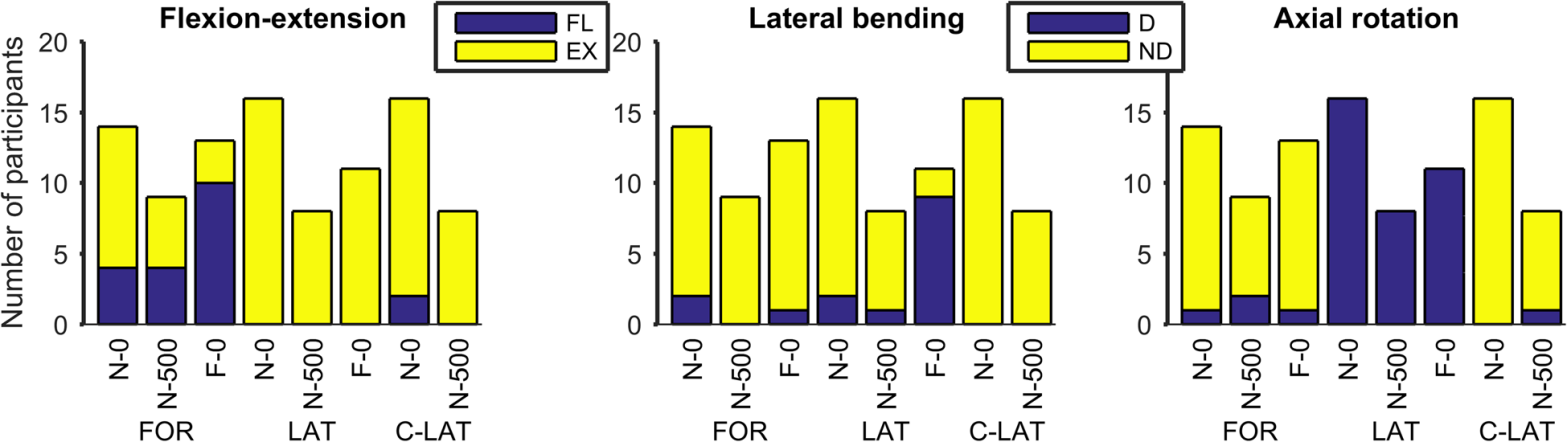
Movement direction of the trunk in DMD patients when performing daily activities. Abbreviations: N = near, F = far, 0 = without weight, 500 = 500 g object, FOR = forward reaching, LAT = reaching laterally, C-LAT = reaching contra-laterally, FL = flexion, EX = extension, D = towards dominant side, ND = towards non-dominant side

The direction of movement was largely the same for all DMD participants (Fig. 3). The largest variation in movement direction could be seen in flexion-extension when reaching forward. Both flexion and extension movements were made by the DMD participants, while trunk extension was seen in the other tasks. Lateral bending was mainly performed towards the non-dominant side, in other words opposite to where the arm was lifted for reaching, except for far lateral reaching. Axial rotation was performed towards the dominant side when reaching laterally and towards the non-dominant side when reaching forward and contra-laterally. The movement direction for DMD participants was essentially the same as in the HC.

**Fig. 4:**
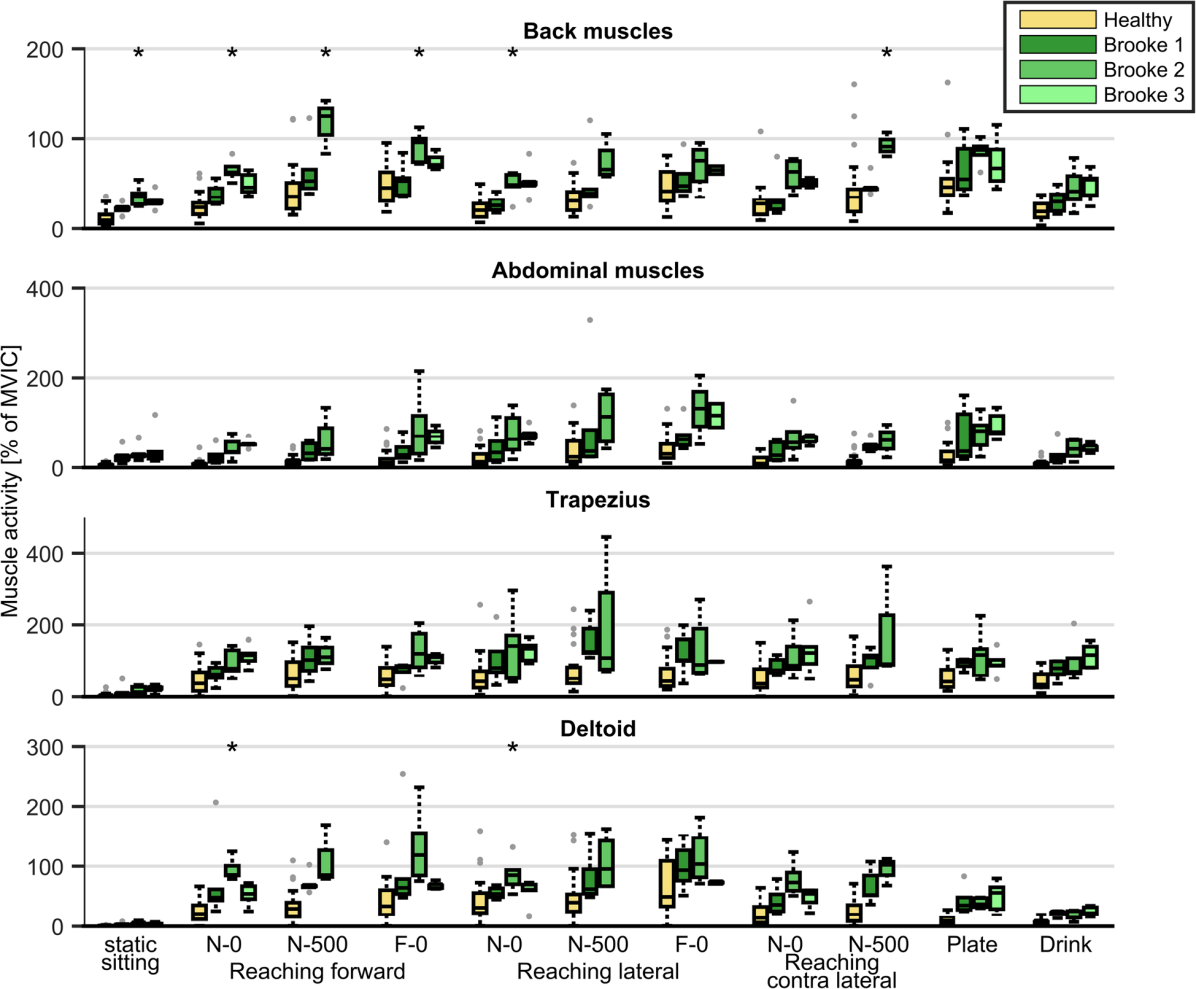
Normalized muscle activity in healthy controls and DMD patients with different Brooke scales. Abbreviations: N = near, F = far, 0 = without weight, 500 = 500 g object, * *p* < 0.05 between Brooke scales

Normalized muscle activity was significantly higher in all muscles and all tasks for DMD patients compared to HC (Fig. 4, Additional file 4). Static sitting (without back or armrests) already required approximately twice as much of trunk muscle capacity in DMD patients than in HC. The ability to perform a task was related to the percentage of muscle capacity used. This could for example be seen when comparing reaching forward without object and with a 500 g object (Fig. 4, Additional file 4). All DMD patients with Brooke scale 1 were able to perform the task with a 500 g object, but only half of the DMD patients with Brooke scale 2 and none of the subjects with Brook scale 3 could. However, those patients with Brooke scale 2 needed around 100% of their back and arm muscle capacity to execute the task.

## Discussion

This study provides new insights in the role of trunk movements and used muscle capacity in DMD patients when performing seated tasks. During arm tasks the trunk shows a larger range of motion in DMD patients compared to healthy controls, combined with increased normalized trunk muscle activity. This reflects that due to compensatory movement, demands on trunk muscles are increased which is compounded by trunk muscle weakness.

Both maximum active trunk ROM and maximum trunk joint torque were significantly decreased in DMD patients compared to HC, indicating that their overall trunk capacity is already less compared to HC. Although this finding is not surprising, this is the first study to show it in a quantitative manner. However, the limitations found in maximum ROM are unlikely to result in restrictions when performing tasks such as tested here, because the maximum trunk ROM (Fig. 1) was less than generally used to perform daily tasks (Fig. 2).

Interestingly, we found that boys in early disease stages (e.g. Brooke scale 1) already showed lower trunk joint torque compared to HC. Additionally, trunk joint torque (in Nm) did not significantly decrease with Brooke scale, while shoulder abduction torque did. The latter was also found in previous research [4]. This could indicate that arm function (i.e. Brooke scale) is decreasing first or that the decrease in trunk function is independent of the decrease in arm function. However, as bodyweight increases with age, and joint torque does not increase with body weight, function decreases [15]. Indeed, when we corrected trunk joint torque for body weight in DMD patients, we found a significant decrease with Brooke scale, implying that functional trunk strength does decrease with disease stage.

Increased trunk lateral bending and/or flexion-extension was found in DMD patients in all tasks. It is remarkable that this was even found when reaching within arm length distance, since the reaching distance could be shorter for patients. DMD patients likely reduce shoulder and upper arm muscle activity using increased trunk lateral bending towards the non-dominant side to reduce shoulder flexion and abduction. By leaning towards the non-dominant side, the dominant shoulder and arm are automatically positioned higher so less shoulder muscle effort is needed to lift the arm for reaching. Opposite to what was initially expected, the increased ROM in trunk flexion-extension was mainly in extension direction. This could mean that the DMD participants lean backwards in order to keep balance, as is also seen in patients with spinal cord injuries [16], or that patients extend their spine from an initially more slumped posture. This also positions the shoulder higher to reduce shoulder muscle effort and allows for a greater ROM of the shoulder [17].

These compensatory trunk movements are likely crucial to accomplish a task when arm function is insufficient. Compensatory trunk movements are also seen in children with cerebral palsy when performing daily tasks and were related to decreased upper extremity function [18, 19]. Unexpectedly, we did not find a significantly larger increase in compensatory trunk movements with task difficulty (e.g. object weight) in DMD patients compared to HC. It could be that patients already use the most optimal strategy in the easiest tasks (e.g. reaching nearby without weight) and therefore further increasing trunk movements is not beneficial. Alternatively, trunk function could limit increasing the compensatory movements as muscle activity levels did approach the maximum values. However, the median change in trunk ROM was often twice as high in DMD patients compared to HC. It is therefore also possible that we did not find a significant increase due to lack of statistical power, also due to the fact that DMD patients with less good arm function could not perform the more difficult task. No significant differences were found in trunk ROM between patients with different scores on the Brooke scale, although it was expected that compensatory trunk ROM would increase with Brooke scale. This is likely caused by small numbers of subjects in all categories.

Normalized muscle activity was significantly higher in patients with DMD compared to HC for all muscles and all tasks. Normalized muscle activity also increased until the task could not be performed. Despite possible overestimation due to non-maximum MVIC, we found that normalized back muscle activity was around 100% when the maximum arm muscle (e.g. deltoid and trapezius) activity was reached. This indicates that back muscle function plays a more important role than thought, so the arm might not be the only limiting factor accomplishing tasks. It is likely that compensatory trunk movements are limited by increasing back muscle activity with disease progression, due to which patients lose the ability to accomplish the task.

The percentage of trunk muscle capacity used when sitting upright was already two times higher in patients with Brooke scale 1 compared to HC and this normalized activity level is even higher when performing tasks. This indicates early trunk muscle weakness in relation to motor function, which contrasts with previous studies indicating that trunk function is good in the ambulatory phase [5, 6]. When a higher percentage of the maximum muscle capacity is used, this leads to faster development of fatigue and possibly to overloading of the muscles [20]. Clinicians should take this increased muscle activity into account for function assessment and development of interventions. Proper seating, back rests or the use of other trunk supportive devices can reduce trunk muscle fatigue during the day [21]. However, it is important that patients are still able to move their trunk, despite increased activity, to accomplish tasks independently. Also physical muscle strength training might reduce fatigability [22].

There are several limitations to this study. The sample size was small when subcategorizing the DMD patients based on Brooke scale. Therefore, the power to detect differences in trunk ROM between these categories may have been too low. Furthermore, only patients with relatively good arm function could perform the more difficult tasks, which reduced statistical power. The control group was not completely matched with the DMD patients in terms of gender. However, there were no significant differences between boys and girls in the HC group. The normalized trunk muscle activity was based on standardized seated MVIC tasks, which probably does not correspond to the actual maximal values for trunk muscle activity. As a consequence, 100% muscle activity does not necessarily correspond to the maximum capacity, but is likely an overestimation. However, since the MVIC tasks were standardized across all participants, it showed that DMD patients used significantly more muscle activity compared to healthy subjects. Reaching distances were based on the distances that could be reached without moving the trunk. Consequently, the reaching distances varied between subjects and groups. In general, patients with weaker arm muscles reached towards shorter distances, however even though the distance was shorter they showed increased trunk movement compared to HC. Lastly, as described before [10], reaching distance and height were set based on subjects’ sitting posture. Small changes in posture could already influence the distance and height and cause variability between tasks within and between subjects. Since we were interested in self-selected movements of the trunk, we did not choose to standardize sitting posture.

## Conclusion

Trunk capacity (joint torque and active ROM) is reduced in DMD patients compared to HC. They used compensatory lateral bending and trunk flexion-extension movements to accomplish daily tasks, in combination with increased normalized muscle activity. The compensatory movements did not significantly increase more with task difficulty (e.g. increasing object weight) compared to HC and also did not increase with Brooke scale, although differences could be seen. Percentage of muscle capacity used was higher in patients with DMD for all muscles and in all tasks, which could result in early development of muscle fatigue. Clinical interventions are necessary to reduce the muscle fatigue, like development of dynamic assistive devices or implementing proper seating. However, (compensatory) trunk movements should not be restricted because this will likely lead to limitations in accomplishing tasks independently.

## Additional files

Additional file 1: Maximum trunk angles. Median and interquartile ranges for maximum trunk angles during active trunk movements in healthy controls, DMD participants and DMD participants sorted by Brooke scale category. (XLSX 11 kb)

Additional file 2: Joint torques. Median and interquartile ranges of the measured joint torques in healthy controls, DMD participants and DMD participants sorted by Brooke scale category. (XLSX 11 kb)

Additional file 3: Trunk ROM. Median and interquartile ranges trunk ROM when performing daily tasks in healthy controls, DMD participants and DMD participants sorted by Brooke scale category. (XLSX 15 kb)

Additional file 4: Normalized muscle activity. Median and interquartile ranges of normalized muscle activity when performing daily tasks in healthy controls, DMD participants and DMD participants sorted by Brooke scale category. (XLSX 16 kb)

## Abbreviations

DMD: Duchenne muscular dystrophy
HC: Healthy controls
MVIC: Maximum voluntary isometric contractions
ROM: Range of motion
sEMG: Surface electromyography
